# Molecular regulation of electrolytes for enhancing anode interfacial stability in lithium–sulfur batteries†

**DOI:** 10.1039/d3cc01179h

**Published:** 2023-06-06

**Authors:** Tianhong Zhou, Yan Zhao, Patrick W. Fritz, Timur Ashirov, Dominika Baster, Mario El Kazzi, Ali Coskun

**Affiliations:** a Department of Chemistry, University of Fribourg, Chemin de Musee 9 Fribourg 1700 Switzerland ali.coskun@unifr.ch; b Electrochemistry Laboratory, Paul Scherrer Institut Villigen 5232 Switzerland

## Abstract

We addressed the poor interfacial stability of the Li metal anode in Li–S batteries through molecular regulation of electrolytes using arylthiol additives with various numbers of anchoring sites. The dual functional tetrathiol additive markedly enhanced the Li anode interfacial stability, controlled the sulfur redox kinetics and suppressed side reactions towards polysulfides, thus leading to an improved capacity retention of 70% after 500 cycles at 1 C.

## Abstract 2

We addressed the poor interfacial stability of the Li metal anode in Li–S batteries through molecular regulation of electrolytes using arylthiol additives with various numbers of anchoring sites.

## Graphical abstract

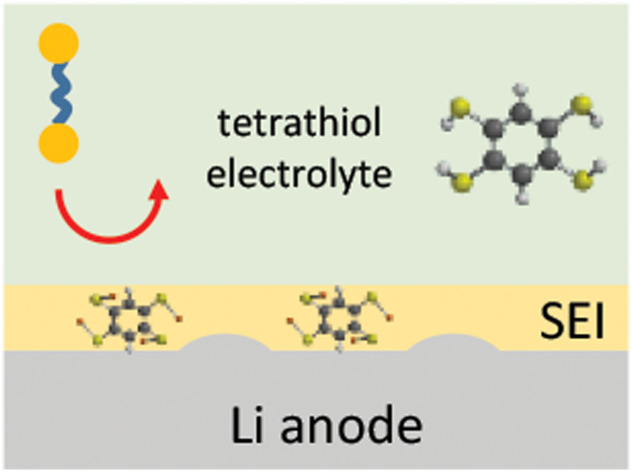

Lithium-ion batteries (LIBs) have played a pivotal role in the advance of portable electronics, electrical vehicles and grid-scale energy storage systems since their commercialization by Sony in 1991.^1,2^ However, the energy densities of today's LIBs are about to reach their theoretical limit,^3,4^ and thus cannot satisfy the booming demand for higher energy density systems. Accordingly, new battery technologies beyond conventional LIBs are being actively pursued. In this direction, the lithium–sulfur (Li–S) battery has gained significant attention owing to its high theoretical capacity of 1675 mA h g^−1^ and theoretical energy density of 2600 W h kg^−1^.^5^ In addition, sulfur is among the most abundant elements and offers a low-cost, light-weight and environmentally friendly alternative to Co and Ni-based cathodes.^6^ Despite these advantages, the practical implementation of Li–S batteries has been rather challenging owing to the shuttling of Li polysulfide (LiPS) intermediates, the poor electronic/ionic conductivity of sulfur and the final discharging product (Li_2_S), and the formation of Li dendrites originating from the formation of a poor solid electrolyte interphase (SEI).^7^

Several strategies have been implemented to mitigate the LiPS shuttling effect and to improve the conductivity of sulfur by integrating various conducting host materials,^8^ designing catalysts to regulate the redox kinetics of sulfur species,^9–11^ optimizing the electrolyte^12,13^ and modifying the separator.^14,15^ These strategies, however, do not address the compatibility problem with the Li metal anode, which is an essential component to realize high energy density. The side reactions between the highly reactive Li anode and LiPS induce serious internal short circuits and thermal runaway issues.^16^ In order to tackle these issues, various design principles have been studied for the Li–S battery electrolytes,^17,18^ which have focused on the use of different solvents to tune the solubility of polysulfides in the electrolyte or alter their redox pathways, and the selection of salts and additives to form the protective layer on the electrode surface, restraining polysulfide shuttling on the sulfur cathode and suppressing the side reactions between the polysulfides and lithium metal.^19^ Electrolyte additives have been commonly applied to form a stable SEI layer and considered as a viable, economical, and efficient approach to overcome the problems originating from the Li metal in Li–S batteries.^20–26^ Dual functional additives or electrolytes, which can simultaneously regulate the redox kinetics of sulfur species and passivate the Li metal surface are, however, rather rare, yet highly desirable for the practical realization of Li–S batteries, considering the easy adaptability of the electrolyte engineering approach to battery manufacturing. In this direction, arylthiols have been shown to be effective to form a stable SEI layer and to control the sulfur redox processes by reacting with sulfur through oligomerization.^27–29^ The latter approach, however, requires a significant amount of organothiol additives (0.15 M) and the H_2_ gas evolution upon reacting with Li metal could also present safety issues at these concentrations. Unlike earlier examples, which showed good results for 1,4 aryldithiol and 1,3,5 aryltrithiol, we reasoned that the 1,2 aryldithiol derivatives could provide efficient interfacial stabilization through the chelating effect. As such, increasing the number of chelating sites could provide efficient interfacial stabilization at lower additive concentrations. Accordingly, herein, 1,2,4,5-aryltetrathiol (tetrathiol) with four SH-groups was designed and synthesized to study the effect of the number and spatial arrangement of sulfur atoms on the SEI chemistry in the Li–S battery. As a control sample, we also tested 1,2-aryldithiol (dithiol) as an electrolyte additive (Fig. 1a). The comparative electrochemical analysis of tetrathiol and dithiol-based electrolytes (1 M LiTFSI in the mixture of 1,3-dioxolane (DOL) and 1,2-dimethoxyethane (DME) (1 : 1 by volume) with 2 wt% LiNO_3_ and ∼0.5 mg mL^−1^ dithiol or tetrathiol) revealed the superior electrochemical performance of the tetrathiol additive for the Li–S battery, which exhibited higher reversible capacity of 483.3 mA h g^−1^ and capacity retention of 70% after 500 cycles at 1 C. The addition of tetrathiol with multiple anchoring sites offers higher electrochemical activity to form more oligomers and generate a highly robust Li_4_-tetrathiol-containing SEI, which contributes to achieving a homogeneous electrochemical stripping and plating process with uniform Li morphology.

**Fig. 1:**
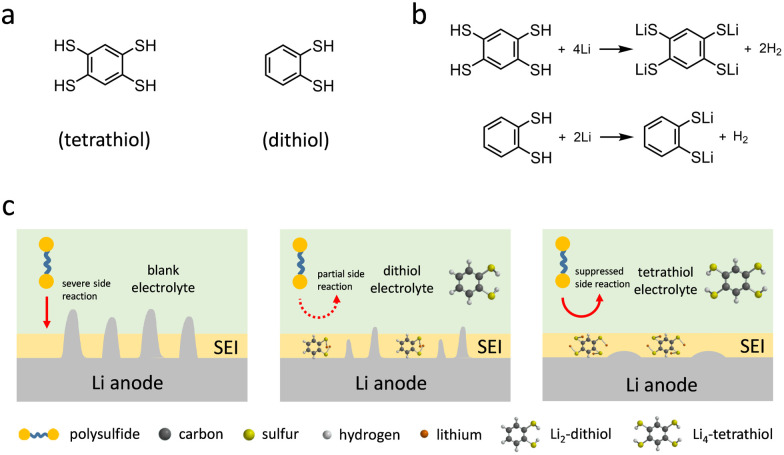
(a) Molecular structures of tetrathiol and dithiol. (b) Reaction between organothiol and Li. (c) The schematics of the Li–S batteries with various electrolytes on the Li anode side.

1,2,4,5-Benzenetetrathiol was synthesized from 1,2,4,5-tetrachlorobenzene in 60% yield and its formation was verified by nuclear magnetic resonance (NMR) spectroscopy analysis (Fig. S1 and S2, ESI†). DFT calculations provided the electron density maps of the highest occupied molecular orbital (HOMO) and lowest unoccupied molecular orbital (LUMO) energies of tetrathiol and dithiol molecules (Fig. S3, ESI†). Remarkably, tetrathiol exhibited a lower LUMO energy level (−1.36 eV) compared to that of dithiol (−1.00 eV), which points to preferential participation in the SEI formation of tetrathiol. Based on the reaction between organothiols and Li,^27^ tetrathiol with four active anchoring sites reacts with four Li, forming lithium benzenetetrathiolate (Li_4_-tetrathiol) and H_2_, whereas dithiol with two anchoring sites reacts with two Li and forms lithium benzendithiolate (Li_2_-dithiol) (Fig. 1b). The schematic illustration for the Li–S battery electrolytes without or with dithiol and tetrathiol additives is shown in Fig. 1c.

The electrochemical performances of tetrathiol, dithiol (∼0.5 mg mL^−1^) and blank electrolytes were evaluated in Li|Li symmetric cells at a high current density of 3 mA cm^−2^ and a capacity of 3 mA h cm^−2^ (Fig. 2a). The voltage polarization of the cell with blank electrolyte increased rapidly to 200 mV after only around 180 h Li plating/stripping, indicating serious side reactions at the Li surface. As for the cell with dithiol containing electrolyte, the overpotential suddenly increased at around 220 h, originating from inhomogeneous Li deposition. Notably, the cell with tetrathiol containing electrolyte can proceed with cycling over 330 h owing to the formation of a compact SEI layer enabled by uniform interactions with the Li surface through four symmetrical sulfur anchoring groups. Electrochemical impedance spectroscopy (EIS) analysis was carried out on the Li|Li symmetric cells to study the kinetic features of the Li interface during cycling in various electrolytes. After the 1st and the 20th cycles at 3 mA cm^−2^ with 3 mA h cm^−2^ (Fig. 2b, c and Table S1, ESI†), the plots were fitted to an equivalent circuit. The charge transfer resistance (*R*_ct_) of the cell with tetrathiol (3.47 Ω and 6.74 Ω) containing electrolyte was found to be lower than those of the cells with dithiol (4.2 Ω and 10.3 Ω) and blank (4.63 Ω and 10.81 Ω) electrolytes, which was attributed to the lower accumulation of dead Li and to the enhanced Li-ion transport kinetics.^30^ In addition, the surface of the cycled Li metal anode in the symmetric cell with tetrathiol containing electrolyte presented a flat and compact morphology without Li dendrites and dead Li (Fig. 2f and Fig. S4c, ESI†), while the cell with dithiol containing electrolyte showed uneven and loosely packed Li grains (Fig. 2e and Fig. S4b, ESI†). In the case of blank electrolyte, the formation of a mossy and corrosive morphology of Li was observed (Fig. 2d and Fig. S4a, ESI†).

**Fig. 2:**
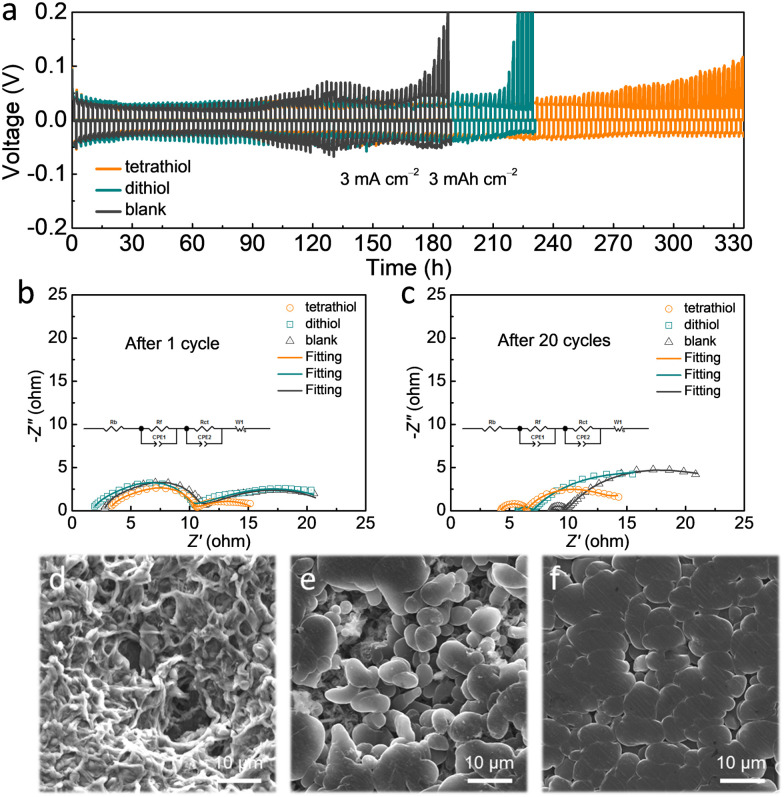
(a) The long-term stability of symmetric cells at 3 mA cm^−2^ with a cut-off capacity of 3 mA h cm^−2^ in various electrolytes. (b and c) EIS curves of the symmetric Li–Li cells with various electrolytes after 1 cycle and 20 cycles. (d–f) SEM images of Li on the Cu substrate with (d) blank, (e) dithiol and (f) tetrathiol electrolytes in symmetric Li–Li cells after 20 cycles.

In order to probe the composition of the SEI, X-ray photoelectron spectroscopy (XPS) analysis was conducted for the cycled Li metal in the Li|Li symmetric cells with different electrolytes after 20 cycles at 3 mA cm^−2^ with 3 mA h cm^−2^ (Fig. S5, ESI†). In the XPS S 2p spectra (Fig. S6, ESI†), we observed two new peaks at 162.6 eV and 162.4 eV in the symmetric cell with tetrathiol and dithiol-based electrolytes, respectively, which is attributed to the organosulfur-containing components of Li_4_-tetrathiol and Li_2_-dithiol^27^ originating from the reaction between organothiol and Li. In the F 1s spectra (Fig. S7, ESI†), the stronger CF_*x*_ signal (688.6 eV) in the case of tetrathiol electrolyte is ascribed to the strong infiltration of the electrolyte to the SEI layer formed with tetrathiol additive.^27^ Therefore, through molecular regulation of electrolytes using organothiol-based additives, a dense and stable SEI formed in the presence of tetrathiol guaranteed the uniformity of Li deposition.

Li–S full cells were assembled to assess the effect of the number and spatial arrangement of SH-groups of the thiol-based additives on the electrochemical performance. The cells with blank, dithiol and tetrathiol electrolytes were cycled at 1 C (1675 mA g^−1^) to evaluate the influence of electrolyte additives (Fig. 3a–d). At the 100th cycle, the discharge capacity of the first plateau of tetrathiol electrolyte was larger than that of dithiol and blank electrolytes (Fig. S8, ESI†), which indicated that apart from the conversion of S to Li_2_S_*x*_ (4 ≤ *x* ≤ 8), more oligomers generated from tetrathiol and S compared with dithiol, and further bonded with Li^+^ to form Li_4_-tetrathiol and Li_2_S.^27^ The reaction of –SH moieties with Li metal and the sulfur cathode leads to a decreased discharge voltage from the 1st cycle to the 100th cycle. After this activation process, a stable interface was formed and the polarization decreased in the following cycles. In addition, the Li–S full cell with tetrathiol requires an activation process of 30 cycles, which suggests that the combination of S and tetrathiol initiates more oligomerization and thus contributes to altering the redox pathways and thus mitigating the shuttling effect. After 500 cycles, the cell with tetrathiol electrolyte exhibited higher reversible capacity of 483.3 mA h g^−1^ and capacity retention of 70%, while the discharge capacity of the cell with blank electrolyte was only 280.3 mA h g^−1^ with the retention of only 41%, due to the continuous side reactions with LiPSs and growth of Li dendrites in the blank electrolyte with a mechanically weak SEI. Although the cell with dithiol electrolyte delivered almost the same capacity as that with tetrathiol electrolyte in the initial 200 cycles, it finally cycled for only 213 cycles, where the charging curve failed to achieve the cut-off voltage. The reproducibility of cells with dithiol and tetrathiol electrolytes has also been demonstrated in Fig. S9 and S10 (ESI†).

**Fig. 3:**
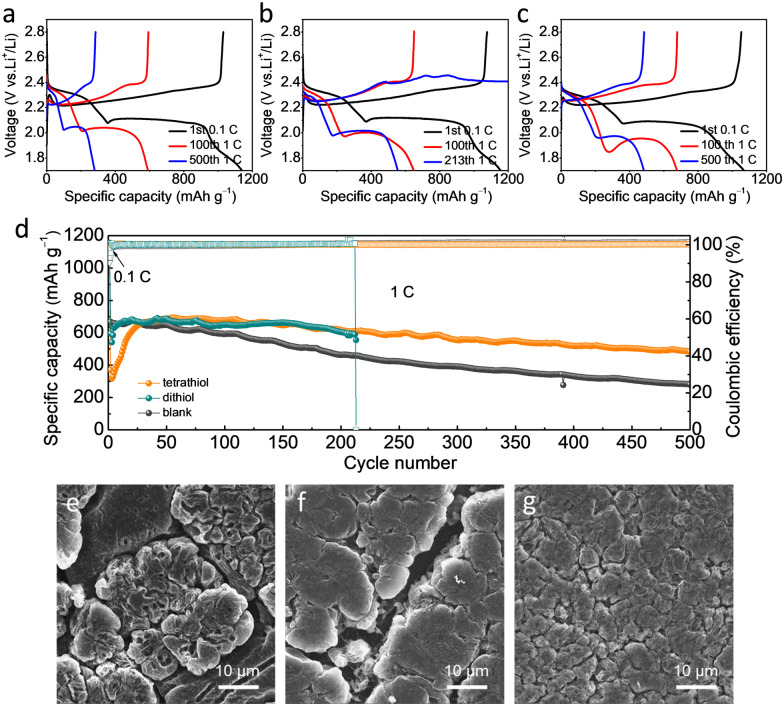
Charge–discharge profiles of the Li–S batteries with (a) blank, (b) dithiol and (c) tetrathiol electrolytes at different cycles. (d) The long cycling performance of Li–S batteries with various electrolytes at 1 C. (e–g) SEM images of the Li anode with (e) blank, (f) dithiol and (g) tetrathiol electrolytes in Li–S batteries after 50 cycles at 1 C.

The cycled Li anodes of Li–S batteries with various electrolytes were disassembled to analyze their morphology. After 50 cycles at 1 C, the Li surface in the blank electrolyte resulted in the growth of irregularly shaped Li with huge cracks (Fig. 3e and Fig. S11a, ESI†). For the electrolyte with dithiol, we observed an uneven morphology with a loose structure having cracks (Fig. 3f and Fig. S11b, ESI†), while a planar and dense surface structure with no apparent cracks was clearly visible in the presence of tetrathiol additive (Fig. 3g and Fig. S11c, ESI†). Therefore, tetrathiol additive with four active anchoring sites forms a more robust SEI, a Li_4_-tetrathiol-containing SEI, which effectively mitigates the side reactions between the Li metal and LiPSs, and greatly reduces the accumulation of dead Li. For the dithiol-based electrolyte, the Li_2_-dithiol-containing SEI can only partly suppress the side reactions on the Li metal anode with LiPSs and electrolyte. In stark contrast, LiPSs can react with the Li anode continuously in the absence of an additive owing to the formation of a poor interface, as evidenced by the mossy and uneven Li anode morphology with obvious cracks. Moreover, the cell with tetrathiol electrolyte showed a superior rate performance compared to the dithiol and blank electrolytes, as shown in Fig. S12 (ESI†). In the view of further practical applications, the cycling performance with high sulfur loading was evaluated with a high *S* mass (3.65 mg cm^−2^) and a low *E*/*S* ratio (10 μL mg^−1^) (Fig. S13 and S14, ESI†). The cell with tetrathiol electrolyte achieved the highest reversible capacity of 669.4 mA h g^−1^ after 70 cycles at 0.1 C. Moreover, a higher sulfur loading of 6.8 mg cm^−2^ with tetrathiol electrolyte (N/P ratio of 2.5; E/S ratio of 4.6 μL mg^−1^) can achieve an initial reversible capacity of 578.1 mA h g^−1^ (∼4 mA h cm^−2^) and maintained a capacity retention of 77% after 40 cycles at 0.05 C (Fig. S15, ESI†). We summarized the detailed parameters of the cells with different sulfur loadings in Table S2 (ESI†). Based on these results, it can be concluded that tetrathiol additive with four centrosymmetric –SH functional anchoring groups is more superior to dithiol one in protecting the Li metal anode for Li–S batteries.

In summary, a new arylthiol-based electrolyte additive has been introduced for Li–S batteries. By optimizing the number and spatial arrangement of anchoring SH-groups, a dense and uniform morphology of the Li anode was obtained to effectively mitigate the side reactions between Li metal and Li-polysulfides. A higher number of anchoring sites have been shown to be the key to decreasing the amount of additive while achieving excellent cycling stability. This work sheds new light on the design principles of electrolyte additives by tuning the number and spatial arrangement of anchoring sites.

A.C. acknowledges the support from the Swiss National Science Foundation (SNF) for funding of this research (200021-188572).

## Conflicts of interest

The authors declare no conflicts of interest.

## Supplementary Material

CC-059-D3CC01179H-s001
