# Supplementary material for: Molecular regulation of electrolytes for enhancing anode interfacial stability in lithium–sulfur batteries
Source: Chem Commun (Camb). 2023 Jun 6;59(53):8286–9. doi: 10.1039/d3cc01179h (PMC10309021; doi:10.1039/d3cc01179h)
Supplement: CC-059-D3CC01179H-s001 [file CC-059-D3CC01179H-s001.pdf]

Electronic Supplementary Material (ESI) for Chemical Communications.
This journal is © The Royal Society of Chemistry 2023

Chemical Communications

Molecular Regulation of Electrolytes for Enhancing Anode Interfacial Stability in Lithium-Sulfur Batteries

*Tianhong Zhou†, Yan Zhao†, Patrick W. Fritz†, Timur Ashirov, Dominika Baster, Mario El Kazzi and Ali Coskun**

T. Zhou, Y. Zhao, P. W. Fritz, T. Ashirov, Prof. A. Coskun
Department of Chemistry, University of Fribourg, Chemin de Musee 9, Fribourg 1700, Switzerland.
E-mail: ali.coskun@unifr.ch

Dr. D. Baste, Dr. M. E. Kazzi
Electrochemistry Laboratory, Paul Scherrer Institut, Villigen 5232, Switzerland.

[†] These authors contributed equally to this work.

Electronic Supplementary Information

Materials

Anhydrous N,N-dimethylacetamide (99.8%), sodium (99.8%), methyl-tertbutylether (99%), 1,2,4,5-tetrachlorobenzene (98%) and sodium 2-propanethiolate (90%) were purchased from Sigma Aldrich. Methyl-tertbutylether (99%) was purchased from Thermo Scientific. Magnesium sulfate (reagent grade), methanol (99%), and hydrochloric acid (37%) were purchased from Fisher Chemicals. All chemicals and solvents were purchased and used without any further purification. The high mass loading sulfur cathode (~3.65 mg cm^{-2}) was ordered from Guangdong Canrd New Energy Technology.

1. Experimental procedures

Synthesis of 1,2,4,5-benzenetetrathiol (tetrathiol) (1): The procedure was adapted from literature.[1]

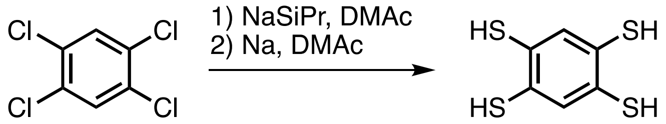

An argon flushed 250 mL two-neck flask was charged with 1,2,4,5-tetrachlorobenzene (1.98 g, 9.17 mmol, 1.00 eq.) and 40 mL dry *N,N*-dimethylacetamide (DMAc). Then, sodium 2-propanethiolate (10.00 g, 90%, 91.71 mmol, 10.00 eq.) was added under argon counterflow. The obtained yellow suspension was heated to 100 °C overnight. Subsequently, sodium (3.30 g, 143.5 mmol, 15.65 eq.) was added in small portions before adding 20 mL of DMAc under vigorous stirring at this temperature resulting in immediate gas formation. Stirring at 100 °C was continued for 8h before carefully adding another portion of sodium (3.30 g, 143.5 mmol, 15.65 eq.) and another 10 mL of DMAc. After stirring overnight, more sodium (3.30 g, 143.5 mmol, 15.65 eq.) and another 20 mL of DMAc were added. After 8h the reaction mixture was cooled to room temperature and the suspension was carefully quenched with isopropanol while cooling with an ice bath (excess sodium can remain in the suspension making directly quenching with water dangerous). Subsequently, the solution was poured onto water

(200 mL) and 200 mL of methyl-tertbutylether (MTBE) was added. The solution was carefully acidified with 5N HCl till a pH<1 was obtained. The layers were separated, and the aqueous phase was extracted twice with 200 mL MTBE. The combined organic layers were washed with water and dried over $MgSO_4$. The solvent was removed, and the obtained crude solid was suspended in 150 mL methanol and stirred for 1 hour. The suspension was filtered, washed with little methanol, and dried under vacuum to give 1,2,4,5-Benzeneteterathiol (1.15 g, 9.17 mmol, 60.8%) as a light-yellow solid. ^{1}H NMR (400 MHz, Chloroform-*d*) δ 7.41 (s, 2H), 3.69 (s, 4H). ^{13}C NMR (100 MHz, Chloroform-*d*) δ 132.9 (4C), 130.0 (2C).

Preparation of tetrathiol and dithiol electrolytes: Tetrathiol was dissolved in a blank electrolyte and stirred for 3 h. The suspension was filtered to obtain a saturated light-yellow tetrathiol electrolyte (~0.5 mg mL^{-1}). The same concentration of dithiol electrolyte was prepared and stirred for 3 h.

2. Material characterizations

Liquid phase NMR spectra were recorded on a Bruker Ascend 400 MHz spectrometer using deuterated solvents. Density functional theory (DFT) calculations of HOMO-LUMO energy levels was performed on Spartan software with a basis set of equilibrium geometry at ground state and B3LYP/6-311++G**. The morphologies of Li metal anode were observed by the field emission scanning electron microscopy (FE-SEM, Tescan Mira3 LM FE). The compositions of SEI were characterized by X-ray photoelectron spectroscopy (XPS) with a VG ESCALAB 220iXL spectrometer (Thermo Fisher Scientific) using focused monochromatized Al Kα radiation (1486.6 eV) with a beam size of ~500 μm^2 (power of 150 W). All the samples were rinsed with anhydrous DME to remove the Li salts prior to their analysis.

3. Electrochemical characterizations

The batteries were assembled in a glove box using CR2032-type coin cells with Celgard 2400 as the separator and the electrolyte amount of 40 µL was used for each coin cell except the high-loading Li-S battery. The blank electrolyte was 1 M LiTFSI in the mixture of 1,3-dioxolane (DOL) and 1,2-dimethoxyethane (DME) (1:1 by volume)

with 2 wt% $LiNO_3$. All battery performance tests were done using a Land 2001A battery system.

Electrochemical Impedance Spectroscopy (EIS) measurements were conducted via VMP3 electrochemical workstation (Bio Logic Science Instruments), wherein the voltage amplitude was set to 5 mV in the frequency range of 100 kHz to 10 mHz. For the symmetric Li|Li test, cells were first cycled at 50 μA between 0–1.5 V (versus Li^+/Li) for 10 cycles. 15 mAh cm^{-2} Li was predeposited on Cu electrode at 0.5 mA cm^{-2}, then cycled at 3 mA cm^{-2} for 1 h. For the Li-S battery, sulfur was first melted into CMK-3 (sulfur@CMK-3) at 155 °C for 12 h by weight ratio of 7:3. The cathode was then prepared by mixing sulfur@CMK-3, Super-P and polyvinylidene difluoride (PVDF) in N-methyl-2-pyrrolidone (NMP) in weight ratio of 8:1:1. The areal loading of sulfur was about 1 mg cm^{-2}.

1. Supporting figures and tables

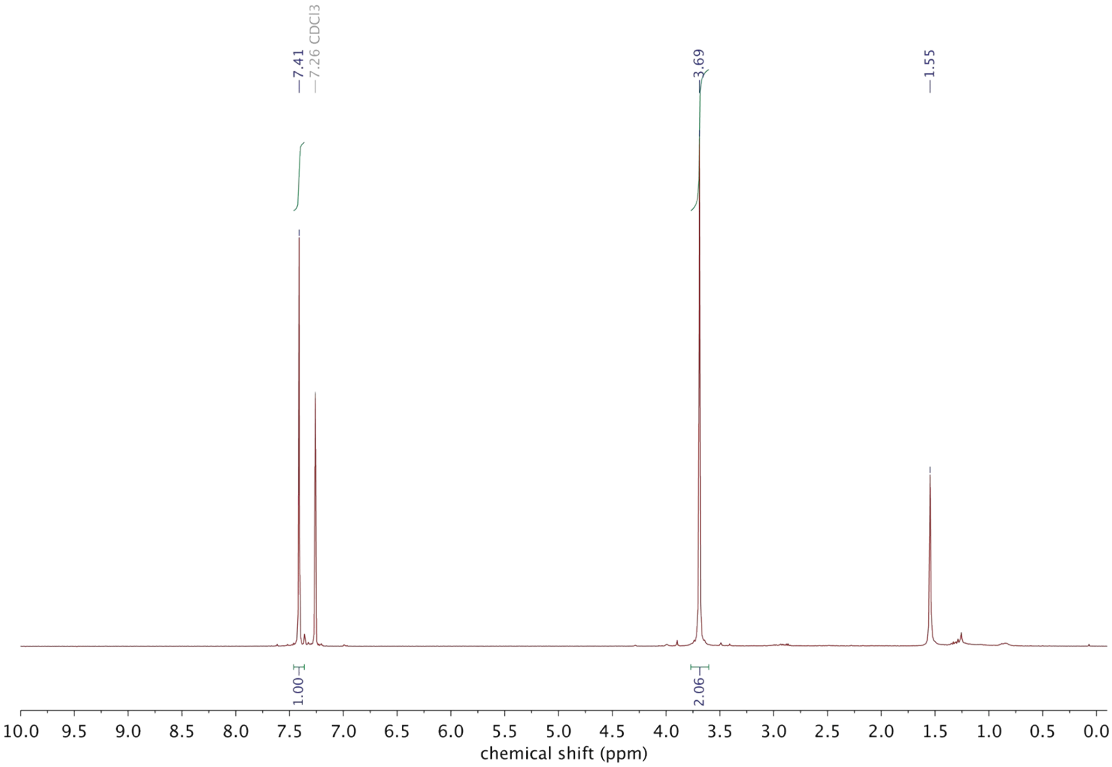

Figure S1. ^{1}H NMR (400 MHz, $CDCl_3$, 298 K) spectrum of tetrathiol.

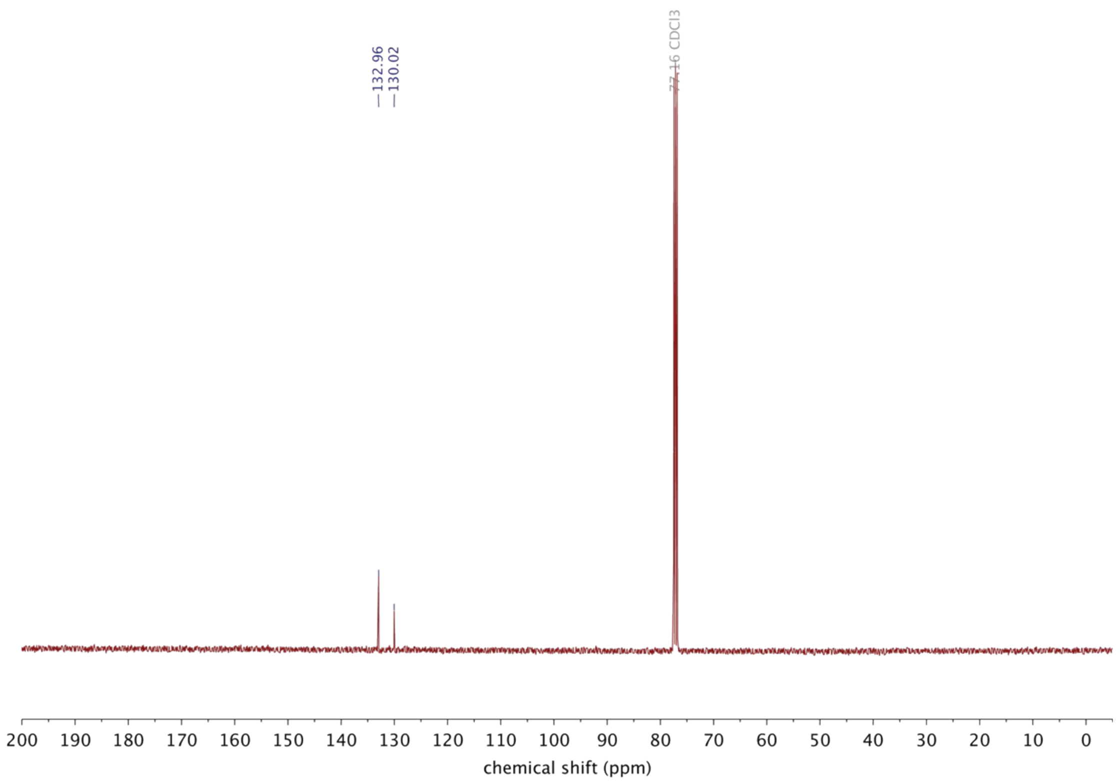

Figure S2. ^{13}C NMR (100 MHz, $CDCl_3$, 298 K) spectrum of tetrathiol.

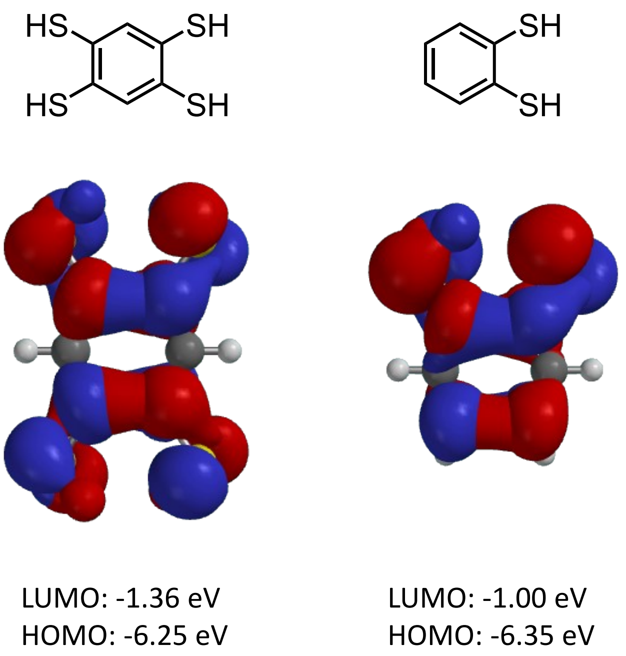

Figure S3. The HOMO and LUMO energy levels of tetrathiol and dithiol.

Table S1. Fitting results of the equivalent circuit displayed in Figure 2b and 2c.

	After 1 cycle			After 20 cycles		
	blank	dithiol	tetrathiol	blank	dithiol	tetrathiol
R_b (ohm)	2.56	1.53	3.05	8.52	5.68	4.24
R_f (ohm)	2.55	3.19	3.26	1.18	1.30	2.21
R_{ct} (ohm)	4.63	4.20	3.47	10.81	10.3	6.74

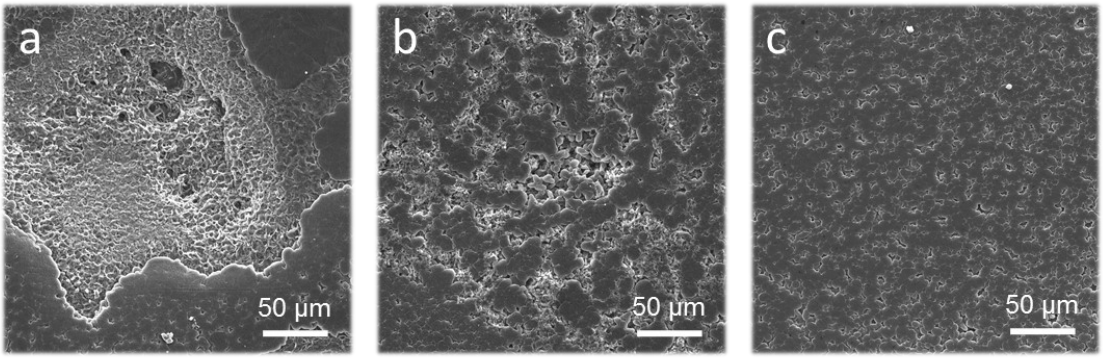

Figure S4. Low magnification SEM images of Li on Cu substrate with (a) blank, (b) dithiol and (c) tetrathiol electrolytes in symmetric Li-Li cells after 20 cycles.

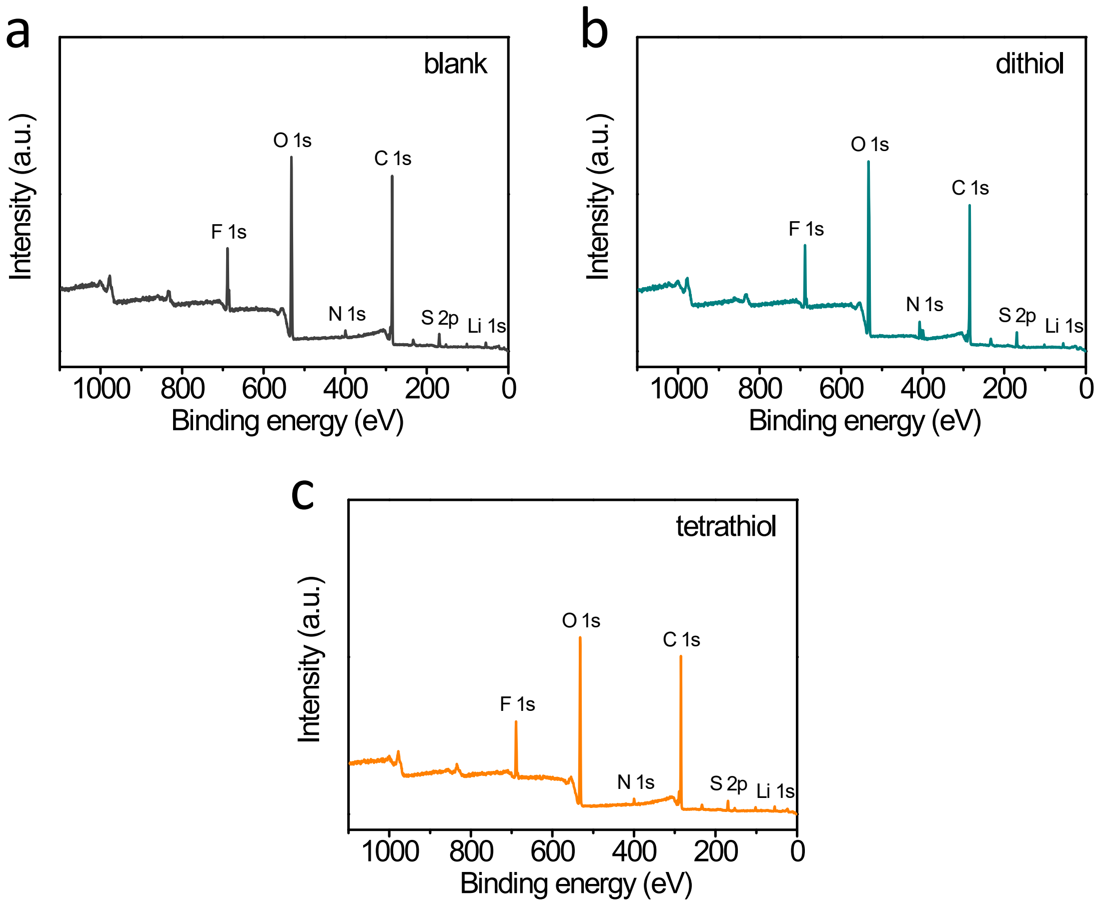

Figure S5. Overall XPS survey spectra of SEI in symmetric Li-Li cells with (a) blank, (b) dithiol and (c) tetrathiol electrolytes after 20 cycles.

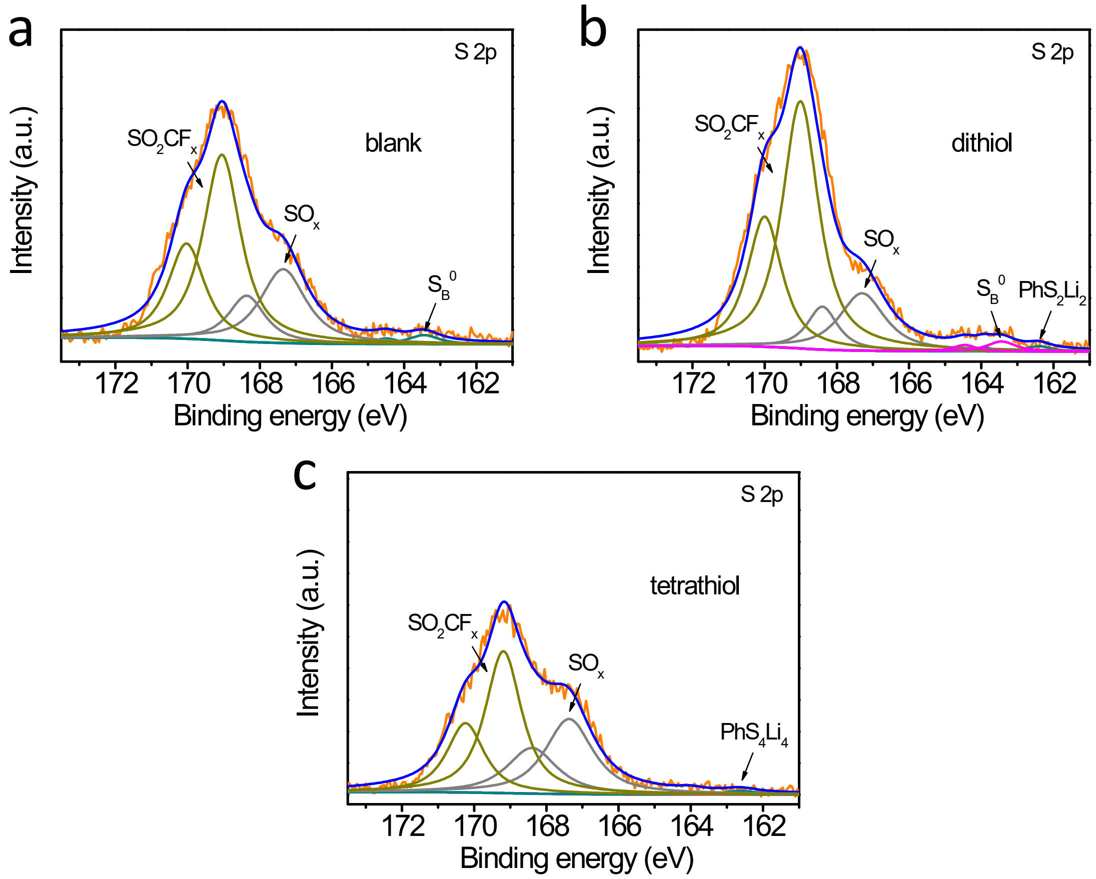

Figure S6. S 2p XPS profiles of SEI in symmetric Li-Li cells with (a) blank, (b) dithiol and (c) tetrathiol electrolytes after 20 cycles.

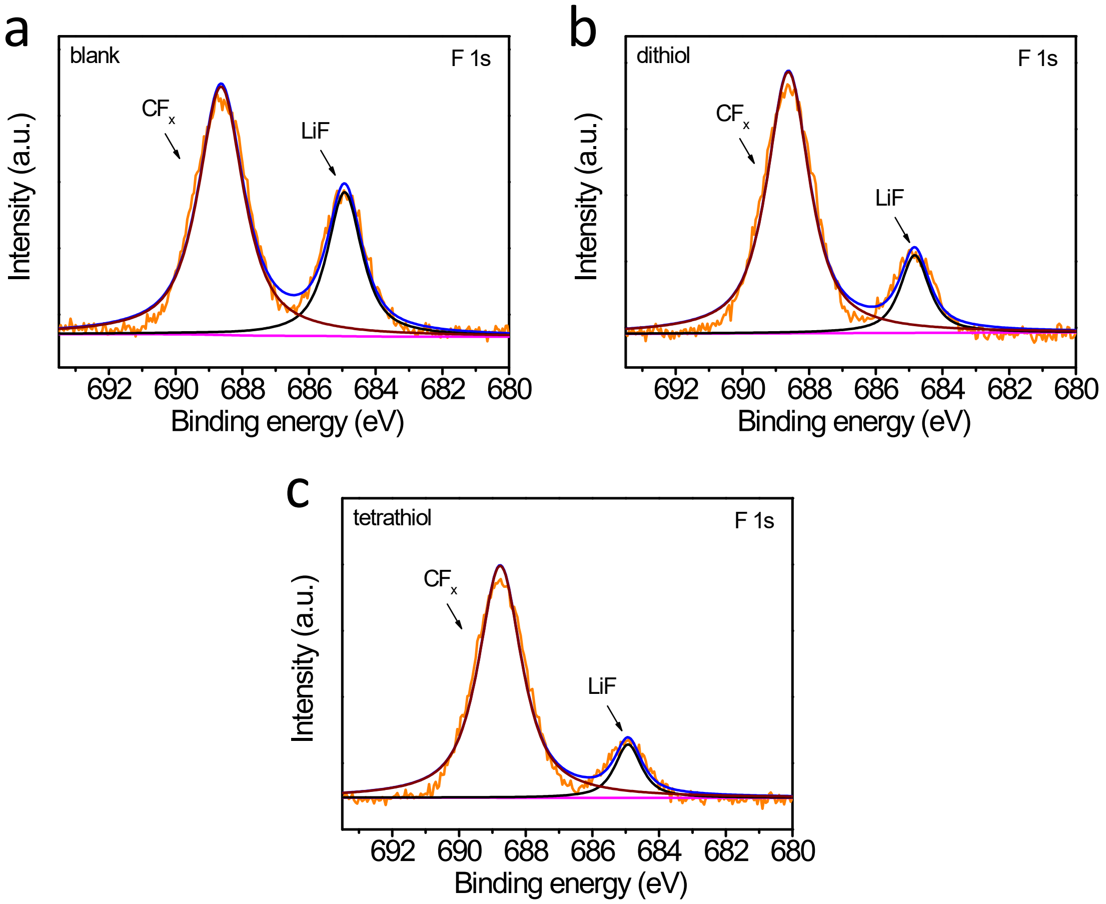

Figure S7. F 1s XPS profiles of SEI in symmetric Li-Li cells with (a) blank, (b) dithiol and (c) tetrathiol electrolytes after 20 cycles.

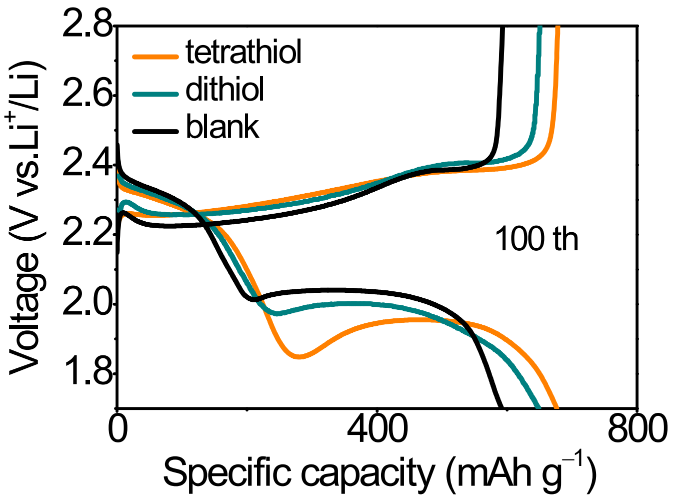

Figure S8. Charge-discharge profiles of Li-S full cell with various electrolytes at 100th cycle at 1 C.

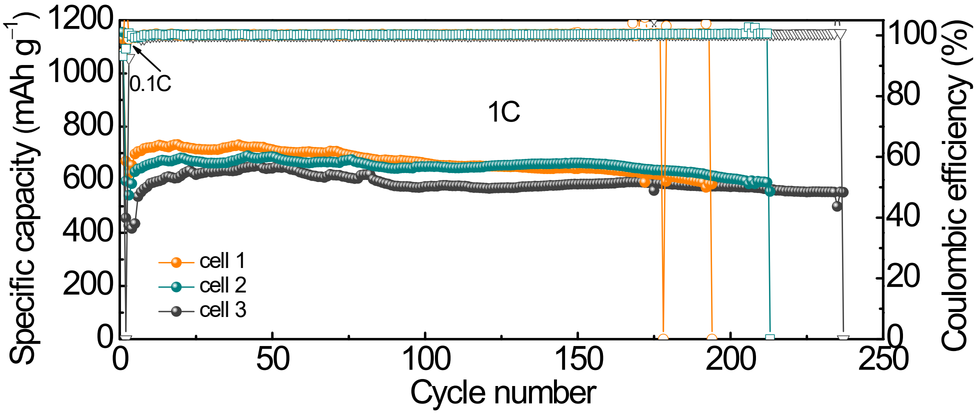

Figure S9. Reproducibility test. Cycling performance and CE at 1 C of Li-S cells with dithiol electrolyte.

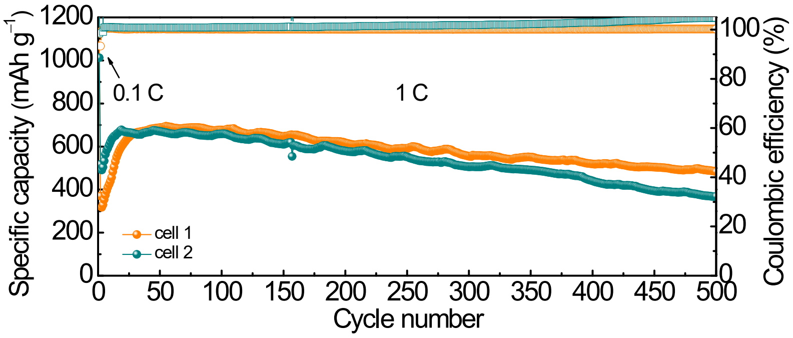

Figure S10. Reproducibility test. Cycling performance and CE at 1 C of Li-S cells with tetrathiol electrolyte.

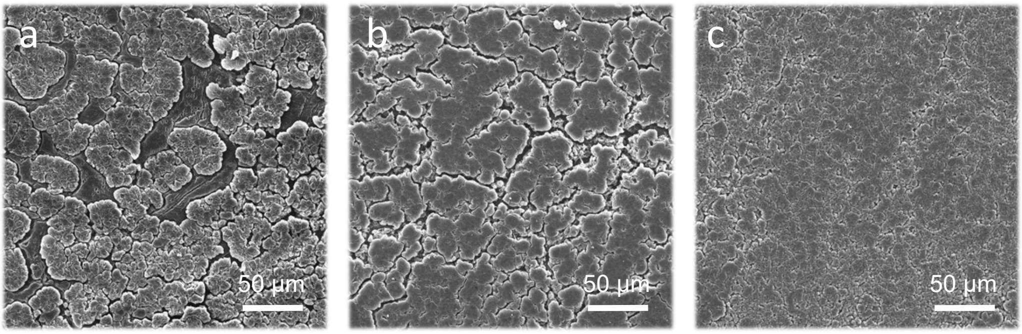

Figure S11. Low magnification SEM images of Li anode with (a) blank, (b) dithiol and (c) tetrathiol electrolytes in Li-S batteries after 50 cycles at 1 C.

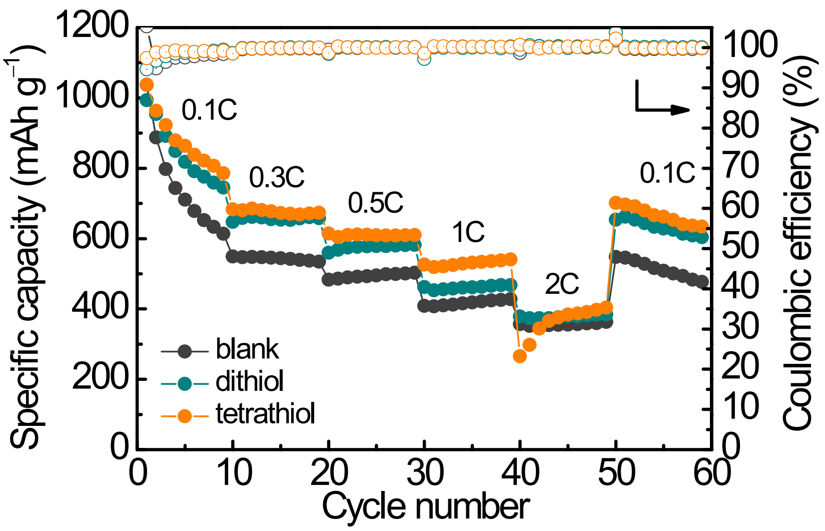

Figure S12. Rate performance of Li-S batteries with various electrolytes.

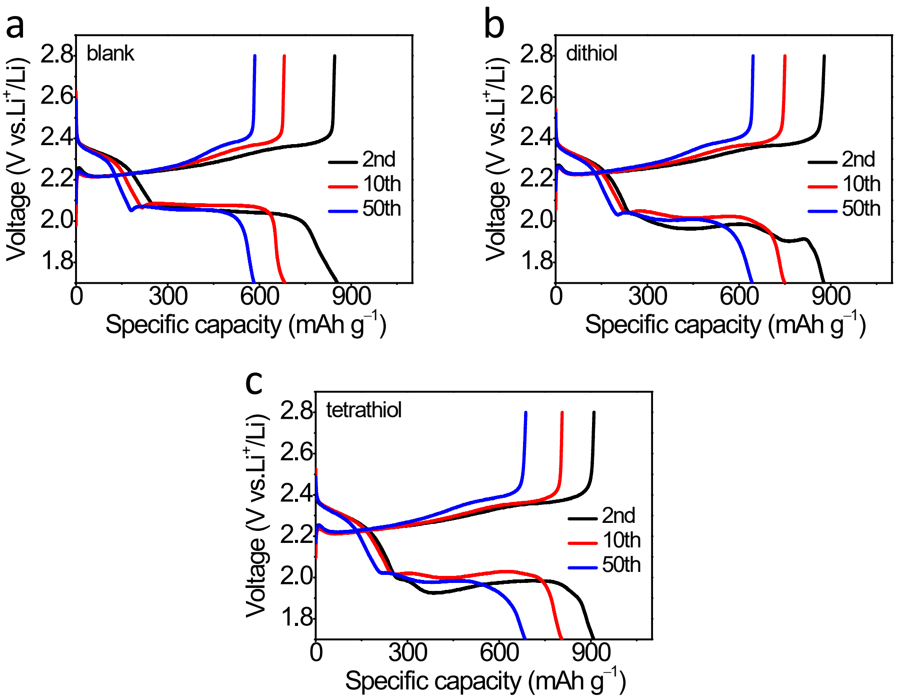

Figure S13. Charge-discharge profiles of the high-loading Li-S batteries at 0.1 C with (a) blank, (b) dithiol and (c) tetrathiol electrolytes at different cycles.

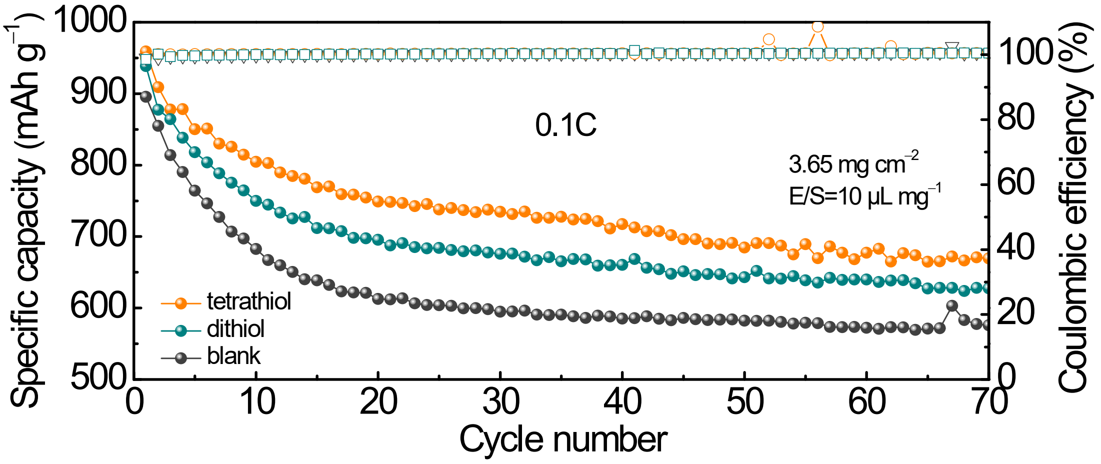

Figure S14. The cycling performance of Li-S batteries with various electrolytes at 0.1 C with a high loading S cathode (3.65 mg cm^{-2}) and a low E/S ratio (10 μL mg^{-1}).

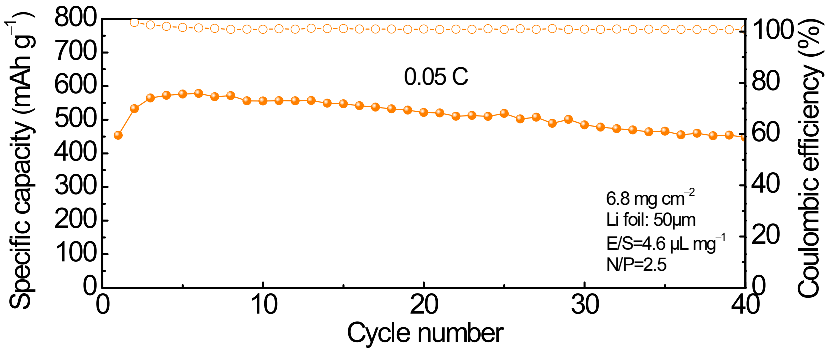

Figure S15. The cycling performance of Li-S battery with tetrathiol additive at 0.05 C with a high loading S cathode (6.8 mg cm^{-2}), a low E/S ratio (4.6 μL mg^{-1}) and a low N/P ratio (2.5).

Table S2. The parameters of the cell with different sulfur loading.

Loading	Cathode	Anode	The amount of electrolyte
1 mg cm^{-2}	sulfur@CMK(7:3): Super-P:PVDF= 8:1:1	Thick Li foil	40 μL
3.65 mg cm^{-2}	commercial cathode (sulfur@C (8:2): Super-P:(PVDF+ LA133)= 86:4:10)	50 μm Li foil	40 μL
6.8 mg cm^{-2}	sulfur-SWCNTs(7:3) : Super-P: PTFE = 6:2:2 (pressed under 7 ton)	50 μm Li foil	35 μL

References

1. Thomas W. T. Muesmann, A. Mietrach, J. Christoffers and Mathias S. Wickleder, *Zeitschrift für anorganische und allgemeine Chemie*, 2010, **636**, 1307-1312.
